# Supplementary material for: A bivalent CMV vaccine formulated with human compatible TLR9 agonist CpG1018 elicits potent cellular and humoral immunity in HLA expressing mice
Source: PLoS Pathog. 2022 Jun 23;18(6):e1010403. doi: 10.1371/journal.ppat.1010403 (PMC9223316; doi:10.1371/journal.ppat.1010403)
Supplement: S1 Table — (DOCX) [file ppat.1010403.s001.docx]

**Supplemental Table 1**

**Antibodies Resource Table**

| **Antibody** | **Source** | **Catalogue number** |
| --- | --- | --- |
| Anti-Human CD3 (Clone: SK7) APC | BD Biosciences | 340440 |
| PerCP – Cyonine 5.5 Anti-Human CD8a (clone: RPA-TB) | EBioscience | 45008842 |
| FITC Mouse Ant-Human CD4 | BD Pharmingen | 555346 |
| Pacific Blue Mouse Anti-Human CD4 | BD Pharmingen | 558116 |
| Alexa Flour 700 Mouse Anti-Human IFN-γ | BD Biosciences | 557995 |
| Anti-Hu-IL2-PE (clone: MQI-17H12) | EBioscience | 12-7029-42 |
| APC Anti-Human TNFα – MAB 11 | BioLegend | 502912 |
| FITC Mouse Anti-Human CD107a | BD Pharmingen | 555800 |
| APC Hamster Anti-Mouse Cd3e (Clone: 145-2C11) | BD Biosciences | 563066 |
| FITC Rat Anti-Mouse CD4 (Clone: H129-19) | BD Biosciences | 553651 |
| PerCP-Cy 5.5 Rat Anti-Mouse CD8a (Clone: 53-6.7) | BD Biosciences | 551162 |
| BV786 Rat Anti-Mouse CD4 (Clone: GK1.5) | BD Biosciences | 563331 |
| APC Rat Anti-Mouse IL-2 | BD Biosciences | 554429 |
| PE RAT Anti-Mouse IFN-γ | BD Biosciences | 554412 |
| PE Cy7 RAT Anti-Mouse TNF (Clone: MP6-XT22) | BD Biosciences | 557844 |
| PE anti-mouse CD45R/B220 (Clone: RA3-6B2) | BioLegend | 103208 |
| FITC antiMu/Hu GL7 (Clone:GL7) | BioLegend | 144604 |
| Anti-Mu-CD95 (APO-1/FAS) APC (Clone: 15A7) | Invitrogen | 17-0951-82 |
| Ms X Cytomegalovirus | EMD Millipore Corporation | MAB810-500µg |
| Alexa Flour 488 goat ant-mouse Ig (H+L) | Invitrogen | A11029 |
| Goat Anti-Mouse Ig HRP | SouthernBiotech | 1010-05 |
| Goat Anti-Mouse IgA-HRP | SouthernBiotech | 1040-05 |
| Goat Anti-Mouse IgM-HRP | SouthernBiotech | 1020-05 |
| Goat Anti-Mouse IgG1-HRP | SouthernBiotech | 1070-05 |
| Goat Anti-Mouse IgG2a-HRP | SouthernBiotech | 1080-05 |
| Goat Anti-Mouse IgG2b-HRP | SouthernBiotech | 1090-05 |
| Goat Anti-Mouse IgG3-HRP | SouthernBiotech | 1100-05 |
| Mouse IgG ELISpot kit | Mabtech AB | 3825-2A |
